# Hemodynamic Instability during Dialysis: The Potential Role of Intradialytic Exercise

**DOI:** 10.1155/2018/8276912

**Published:** 2018-02-27

**Authors:** Scott McGuire, Elizabeth Jane Horton, Derek Renshaw, Alofonso Jimenez, Nithya Krishnan, Gordon McGregor

**Affiliations:** ^1^Faculty of Health and Life Sciences, Coventry University, Coventry, UK; ^2^Go Fit Lab, Ingesport, Madrid, Spain; ^3^Department of Nephrology, University Hospitals Coventry and Warwickshire NHS Trust, Coventry, UK

## Abstract

Acute haemodynamic instability is a natural consequence of disordered cardiovascular physiology during haemodialysis (HD). Prevalence of intradialytic hypotension (IDH) can be as high as 20–30%, contributing to subclinical, transient myocardial ischemia. In the long term, this results in progressive, maladaptive cardiac remodeling and impairment of left ventricular function. This is thought to be a major contributor to increased cardiovascular mortality in end stage renal disease (ESRD). Medical strategies to acutely attenuate haemodynamic instability during HD are suboptimal. Whilst a programme of intradialytic exercise training appears to facilitate numerous chronic adaptations, little is known of the acute physiological response to this type of exercise. In particular, the potential for intradialytic exercise to acutely stabilise cardiovascular hemodynamics, thus preventing IDH and myocardial ischemia, has not been explored. This narrative review aims to summarise the characteristics and causes of acute haemodynamic instability during HD, with an overview of current medical therapies to treat IDH. Moreover, we discuss the acute physiological response to intradialytic exercise with a view to determining the potential for this nonmedical intervention to stabilise cardiovascular haemodynamics during HD, improve coronary perfusion, and reduce cardiovascular morbidity and mortality in ESRD.

## 1. Introduction

Chronic kidney disease (CKD) has a world-wide prevalence of 5–10%, equating to ~740 million individuals [1]. In the UK alone, approximately 5.9% of the population has advanced CKD at stages 3–5 [2]. The disease is characterized by the inefficiency of the glomerular to maintain fluid homeostasis, resulting in metabolic acidosis through the accumulation of creatinine, urea, and electrolytes [3]. This leads to cardiovascular and hematological complications such as hypertension, reduced arterial compliance, accelerated atherosclerosis, cardiomyopathy, cardiac fibrosis, and anemia [4–7]. It is common for quality of life to be impaired and life expectancy to be reduced [3, 6, 8].

A glomerular filtration rate (GFR) of <15 ml/min^−1^ 1.73 m^2^ is indicative of end stage renal disease (ESRD) whereby patients may have to undergo renal replacement therapy, more specifically haemodialysis (HD), to replace the typical functions of the kidney. Despite HD being critical to survival, it is associated with numerous side effects including lethargy, fatigue, irritable legs, muscle cramps, nausea, vomiting, dizziness, and perpetual systemic inflammation [9]. Moreover, the rapid removal of excess fluid acutely compromises cardiovascular hemodynamics, reducing cardiac output and mean arterial pressure (MAP). Haemodialysis efficacy can be affected by the need to reduce filtration rates or cease HD altogether, leaving patients above their target dry weight [10–13]. In addition, an impaired cardiac output during HD can lead to systemic hypoperfusion and subclinical ischemia. Cerebral, splanchnic, and myocardial hypoxia potentiates acute and chronic cognitive, gastrointestinal barrier and cardiac dysfunction [10–12, 14]. These deleterious effects highlight the need for effective strategies to attenuate hemodynamic compromise during HD. A solution to this problem would likely have a positive impact on quality of life, morbidity, and mortality in ESRD.

Currently, there are limited therapeutic options with which to tackle hemodynamic instability during HD. Pharmacological and nonpharmacological interventions have been proposed, such as Midodrine, arginine vasopressin, lower limb pneumatic compression, cooling dialysate, hemodiafiltration, nocturnal HD, ultrafiltration, and sodium profiling [10, 11, 14–21]. With limited success from these methods, it is paramount that new strategies to counteract hemodynamic compromise during HD be explored, thus maximizing the efficacy of treatment and minimizing the short and long-term risk to patients. The primary acute effect of exercise is an enhanced cardiac output and MAP in response to increased heart rate and left ventricular (LV) stroke volume. Despite cardiovascular and metabolic derangement in ESRD, this hemodynamic response to exercise may also occur during HD. If so, intradialytic exercise may have the potential to restore cerebral, splanchnic, and myocardial perfusion. It is possible that intradialytic exercise, which is accumulating a solid evidence base in support of its efficacy and safety, could offer a viable alternative to current therapies aimed at alleviating hemodynamic instability during HD.

This review aims to characterize the acute effects of HD on cardiovascular hemodynamics and discuss current strategies to counteract these perturbations. Further, the potential for intradialytic exercise to resolve acutely comprised hemodynamics will be explored. We will examine the current evidence relating to the acute physiological response to intradialytic exercise with a view to determining mechanisms by which “normal” cardiovascular hemodynamics might be restored.

## 2. Cardiovascular Risk in End Stage Renal Disease

Cardiovascular disease (CVD) is the most common cause of death in ESRD [5]. Reduced kidney efficiency is linked to a progressive deterioration in cardiovascular health, ultimately leading to heart failure, myocardial infarction, and stroke [23]. Patients with ESRD have a cardiovascular risk far greater than that explained by hypertension or other traditional CVD lifestyle risk factors alone [12]. Indeed, heart failure and sudden cardiac death are the most common causes of death in HD patients as opposed to atherosclerotic coronary disease [21]. Cardiac pathology in ESRD is, therefore, attributed to numerous CKD sequelae including chronic inflammation, hypertension, increased oxidative stress, abnormal renin angiotensin system activation, production of FGF-23, and arrhythmias [3, 5]. This unique cardiovascular phenotype is, in part, linked to HD treatment itself. Repeated bouts of transient myocardial ischemia, mediated by predialysis inflammation and compromised intradialytic hemodynamics, are known to contribute to maladaptive myocardial remodeling with LV fibrosis, hypertrophy, and diastolic stiffening [11, 14, 24]. Myocardial oxygen demand is chronically increased and prolongation of LV depolarization further impairs contractile function [25, 26]. Thus, CKD, in combination with hemodynamic instability during HD treatment, significantly increases cardiovascular risk in patients with ESRD.

## 3. Hemodynamic Instability 

**Figure 1:**
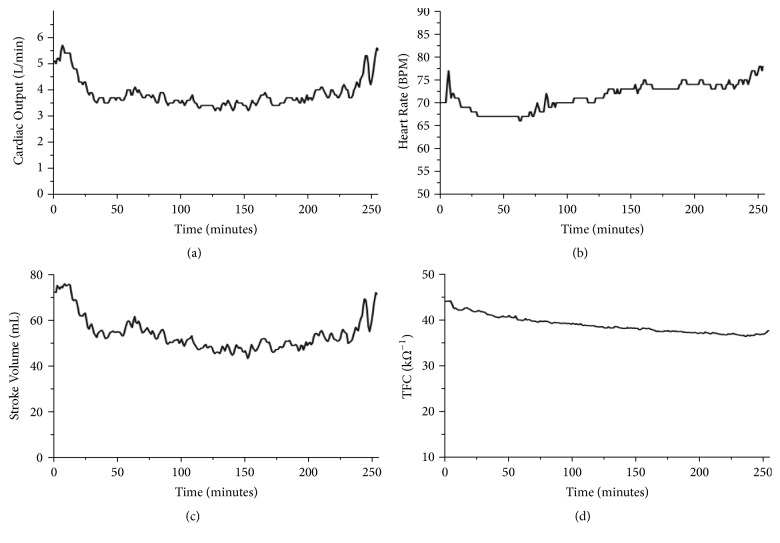
Continuous recording of (a) cardiac output; (b) heart rate; (c) stroke volume; and (d) thoracic fluid content (TFC) during dialysis. Note the decrease in cardiac output/stroke volume and lack of sufficient heart rate compensation [22].

In the absence of a functioning kidney, HD treatment may be initiated to filter waste products and maintain fluid homeostasis. Toxins such as urea, creatinine, and nitrogen are removed, and fluid overload is reversed [3, 15]. However, a large decrease in plasma volume can be problematic during HD [15]. Hemodynamic instability can lead to intradialytic hypotension (IDH) and reduced HD efficacy due to insufficient filtration rates and/or early cessation of treatment [27]. The rapid decline in blood serum volume during filtration has a profound effect on cardiac output (Figure 1). Myocardial preload is impaired by reduced venous return, and contractile force is further compromised by myocardial ischemia [14, 18, 28]. Chronotropic incompetence, which may relate to reduced catecholamine sensitivity because of impaired renal clearance of circulating hormones, has also been observed during HD. The combination of reduced stroke volume and the absence of a compensatory increase in heart rate can prevent the maintenance of appropriate cardiac output [22]. In addition, when large fluid volumes are extracted, there is a delayed reuptake of water from the interstitial space, leading to an inability to normalize arterial plasma volume [29]. In 20–30% of ESRD patients, this cardiovascular milieu corresponds to an overall decline in cardiac output and reduced myocardial and systemic perfusion [11, 17–19]. Ultimately, systemic organ hypoperfusion contributes to the genesis of multiple pathologies.

## 4. Systemic Effects 

Hemodynamic perturbations during HD are known to decrease perfusion of cerebral, splanchnic, and myocardial tissue [10, 11, 14, 30, 31]. It has been reported that impaired intradialytic hemodynamics result in reduced splanchnic region blood flow and ischemic intestinal injury. Consequently, increased gut permeability allows [16, 30] gut flora to “leak” into the circulation, triggering translocation of endotoxin and a proinflammatory environment, clinical features of which can include general malaise and an increased rate of infection [16]. Levels of circulating endotoxin correlate with reduced myocardial contractility and systemic inflammation in CKD [30]. Therefore, ischemia is not localized during HD, rather, there is potential for systemic hypoperfusion. A common complication of HD treatment is postdialytic fatigue which is present in 60–97% of patients [16]. It is speculated that this may be linked to impaired perfusion of the central nervous system as a direct consequence of decreased cardiac output due to hypovolemia and myocardial dysfunction [16]. Patients can need over five hours of sleep to recover from postdialysis fatigue, affecting both HD compliance and quality of life [20]. Ultimately, decreased perfusion of the cerebrum may lead to atrophy of the prefrontal lobe and chronic cognitive impairment [20, 21, 32]. Patients who experience postdialytic fatigue also have a significantly higher incidence of regional myocardial dysfunction [20, 33], further indicating multiorgan hypoperfusion and ischemia.

**Figure 2:**
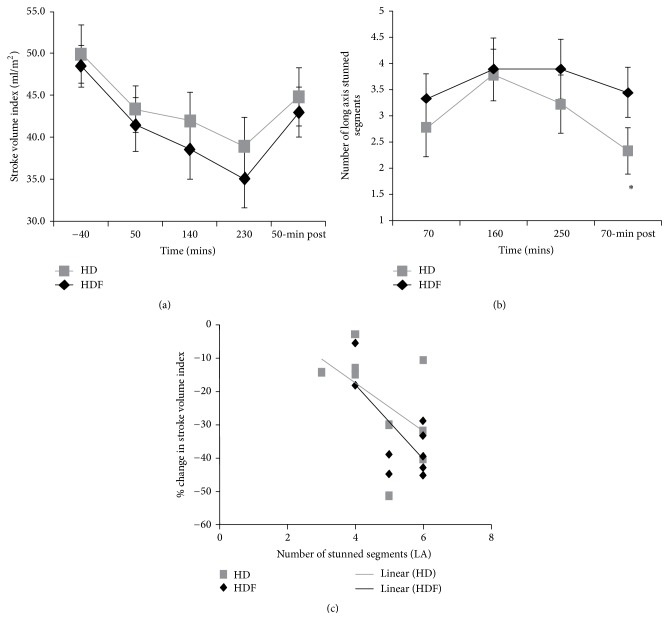
Haemodynamic instability during HD and HDF (a) decreasing stroke volume index identified from aortic flow measurement, with a nadir after 230 mins, and partial recovery at 50 mins after dialysis; (b) number of stunned cardiac segments (long axis) over time (20% reduction from baseline); (c) negative correlation between stroke volume and presence of RWMA [11].  ^*∗*^Significant difference between HD and HDF. LA refers to long axis.

Impaired coronary perfusion, which can result in acute intradialytic myocardial ischemia and stunning, has been extensively documented during HD [6, 10, 11, 14, 17, 18, 20, 24–26, 33–35]. The measurement of regional wall motion abnormalities (RWMA) is commonly used to quantify this phenomenon [10, 11, 14, 21, 24, 26]. Cardiac stunning refers to myocardial segments that present as hypokinetic (reduced ventricular wall/longitudinal thickening), akinetic (no deformation), or dyskinetic (abnormal deformation), whereby a >20% decline in regional cardiac function from baseline is indicative of a stunned segment [10, 26]. In a comprehensive echocardiographic study, nearly half of all assessed myocardial segments developed ischemic RWMA during HD. Furthermore, ejection fraction and systolic blood pressure were acutely reduced [10]. At 12 months, a third of the acutely stunned segments at baseline had progressed to fixed systolic function defects of >60%. The cumulative effect of repeated subclinical myocardial ischemia, therefore, results in maladaptive LV remodeling and permanent LV systolic dysfunction. These findings were confirmed with magnetic resonance imaging (MRI), with which LV contractility and myocardial perfusion were shown to be compromised in 78% of patients [11]. A positive association between long axis RWMA and ultrafiltration volume was observed with both HD and hemodiafiltration (HDF) modalities. Stroke volume progressively declined throughout HD, correlating with the occurrence of ventricular RWMA (Figure 2). In this study, reduced myocardial perfusion was predominantly considered to be a result of reduced microcirculatory blood flow rather than flow in the major epicardial vessels; specific mechanisms are yet to be identified. These data and others provide strong evidence of acute and chronic cardiac dysfunction during and further to HD treatment [6, 8, 9, 11, 17, 18, 24–26, 33, 34]. The investigation of methods to counteract these hemodynamic perturbations appears critical for both patient quality of life and survival. An effective intervention would likely increase MAP and cardiac output during HD, but how this may relate to increased perfusion and reduced ischemic injury is currently unknown.

## 5. Methods to Manage Intradialytic Hemodynamic Instability 

A key measure of hemodynamic instability—intradialytic hypotension—is defined as a drop in systolic blood pressure (BP) of 20 mmHg or a fall in MAP of 10 mmHg during HD. These objective measures are accompanied by symptoms including dizziness, lethargy, and nausea [34]. Intradialytic hypotension is reported to occur in 20–30% of HD treatments [32, 36] and complications include acute hemolysis, air embolus, multiorgan ischemia, pericardial tamponade, bleeding, and sepsis [37]. Strategies to counteract IDH include strict monitoring of fluid/nutritional intake, pharmacotherapy, lower limb pneumatic compression, and different HD modalities [38]. Interventions target one of two mechanisms in the cascade of hemodynamic instability during HD: (1) increasing venous return via vasoconstriction or (2) avoiding a rapid drop in plasma volume. Mixed results have been reported with all these methods, and it appears that medical management of HD related complications is challenging [36].

### 5.1. Pharmacotherapy

Pharmacological strategies to attenuate IDH are limited. Midodrine, an *α*_1_-agonist, may have some efficacy in patients who present regularly with IDH [39]. Activation of the alpha-adrenergic receptors of the arteriolar and venous vasculature increases vascular tone and MAP [39, 40]. This mechanism can improve systolic BP in patients with orthostatic hypotension [41]; however, little benefit was observed in the treatment of IDH [41]. Nevertheless, Midodrine is currently used in clinical practice despite some uncertainty as to its safety and efficacy [39–42]. Arginine vasopressin, (or antidiuretic hormone, ADH), a hypothalamic polypeptide, has also been investigated [43]. Although the action of vasopressin on the convoluted tubule may have little influence on fluid balance in the diseased kidney, vasopressin is a well-recognized vasoconstrictor when bound to the V1*α* receptors in vascular smooth muscle. Its application in IDH has proven somewhat effective [43]; however most studies were of short duration, with small study populations [44].

### 5.2. Pneumatic Compression

Intermittent pneumatic compression of the lower limbs aims to mechanically augment LV contractile force via increased venous return and LV preload [45, 46]. In a randomized crossover trial, air filled compression garments, which circumferentially constricted the lower extremities, had little effect on hemodynamics during HD [46]. More recent data, however, supported the use of this technique, in preference to intradialytic exercise, for the maintenance of MAP and reduction of hypotensive episodes [19]. Neither intervention has been investigated to determine the acute effect on cardiac stunning. Currently, there is insufficient evidence to support the clinical application of pneumatic compression for mitigating hemodynamic instability during HD [19, 45, 46].

### 5.3. Cooling Dialysate

Dialysate fluid typically comprises sodium, potassium, calcium, magnesium, bicarbonate, and glucose which interact with blood flow via a semipermeable membrane. With controlled cooling of dialysate, the associated increase in sympathetic drive has been shown to positively influence MAP and reduce IDH [32, 47]. However, urea compartmentalization may occur when dialysate is cooled, due to increased vasoconstriction of vascular beds [32]. Conversely, the same vasoconstriction of systemic vasculature may aid MAP and prevent dialysis induced vasodilation [32]. Nevertheless, there is the potential for significant patient discomfort, a theoretical risk of hypothermia, and reduced adequacy of dialysis [32, 47]. Despite evidence suggesting a positive therapeutic effect of cooling dialysate, this procedure is not universally adopted [47]. This is likely due to inconclusive evidence of the long-term effects on IDH, and the lack of a consensus regarding optimal cooling procedures [47]. Further investigation into the use of cooled dialysate is warranted.

### 5.4. Hemodiafiltration

Alternative HD modalities such as hemodiafiltration (HDF) utilize pressure gradients to remove solutes over a wider molecular weight range than standard HD [48]. The combination of diffusive and convective dialysis may help prevent IDH by the cooling effect of large convective replacement volumes which can induce greater arterial vasoconstriction and increase MAP [11, 48–50]. This appears to result in greater solute removal, decreased IDH, and reduced mortality and hospitalizations [48]. However, the incidence of RWMA is similar to standard HD [51] suggesting a degree of hemodynamic compromise persists. Evidence to support the use of HDF over standard HD for the prevention of IDH is currently inconclusive [52].

### 5.5. Nocturnal Dialysis

Large reductions in plasma volume during three times weekly (3-4 hrs per session) HD contribute to IDH [53, 54]. As an alternative, nocturnal HD is performed 3–7 times weekly thus avoiding large interdialytic weight gain and hypervolemia [7]. Long-term use has been associated with better intradialytic blood pressure control and solute removal, in addition to reduced LV hypertrophy [54]. However, a meta-analysis of 22,508 patients showed no difference in mortality between home based nocturnal HD and conventional hospital HD [54]. Moreover, 3/4 of nocturnal HD patients were unable to continue treatment due to infection, catheter dysfunction, or ultrafiltration failure [54].

### 5.6. Ultrafiltration and Sodium Profiling

The inability to refill vascular beds during HD may also contribute to IDH [29]. Prolonged HD results in decreased blood plasma volume from impaired reuptake of fluid from the interstitium [29]. Ultrafiltration profiling attempts to avoid large decreases in plasma volume by alternating periods of filtration and recovery to facilitate vascular refilling [29]. Theoretically, when combined with ultrafiltration, dialysate sodium profiling may further increase vascular osmotic pressure, preventing movement of extracellular water from the plasma to the intracellular space [55], and favorably influencing MAP. However, longitudinal data supporting the use of sodium profiling are inconclusive. Additionally, sodium profiling techniques vary considerably in efficacy and each requires further investigation [56]. A recent meta-analysis recommended the use of sodium step wise profiling for clinical practice but acknowledged that more evidence is required to determine the impact on patient outcomes [56].

## 6. Intradialytic Exercise 

**Figure 3:**
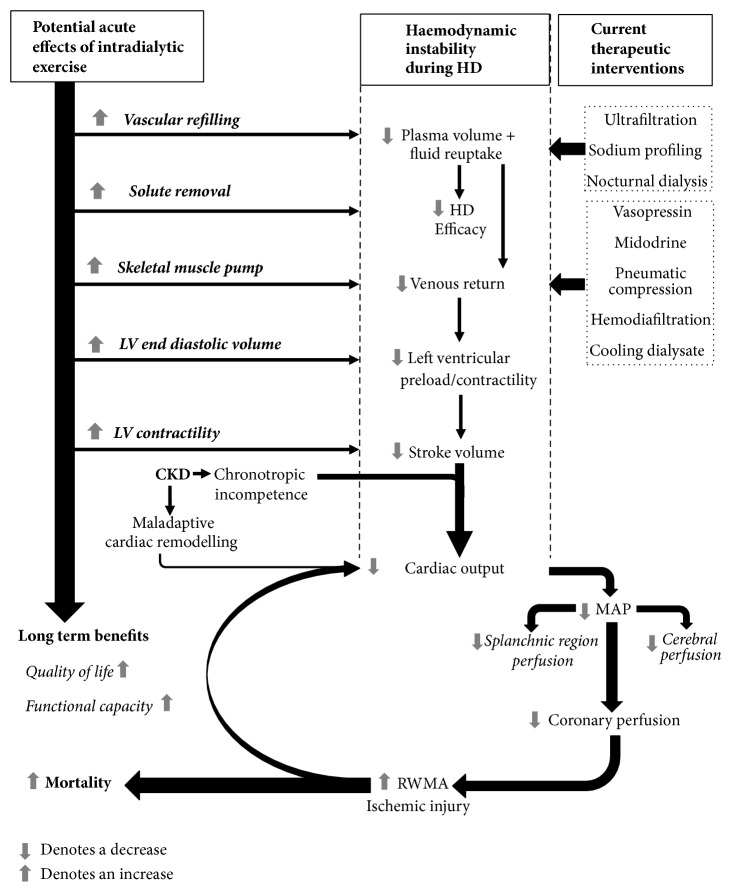
Effects of haemodynamic instability during haemodialysis and mode of action of current therapeutic interventions, and the potential role of intradialytic exercise. HD: haemodialysis, MAP: mean arterial pressure, LV: left ventricular, RWMA: regional wall motion abnormalities, and CKD: chronic kidney disease.

Combating hemodynamic instability during HD is problematic, and it appears that current interventions can be subtherapeutic. To date, studies have not fully investigated the potential for intradialytic exercise to acutely attenuate IDH and its associated outcomes (Table 1). The acute physiological response to exercise in a healthy cardiovascular system is typified by an increased cardiac output achieved through an elevated heart rate and enhanced stroke volume. Sympathetic activation increases heart rate and myocardial contractility leading to higher stroke volume, cardiac output, and arterial pressure. During submaximal exercise, cardiac output can increase fourfold to match the oxygen demand of skeletal muscle [57]. To further increase cardiac output, active skeletal muscle acts upon vascular beds to promote venous return, thus augmenting LV end-diastolic volume and contraction [57]. Arterial vasodilation, supporting oxygen delivery to working muscle, coincides with vasoconstriction in nonactive tissues, meaning cardiac output can be effectively redistributed to the myocardium and skeletal muscle to satisfy the metabolic demands of exercise [58]. It is plausible that this acute response to exercise, specifically increased cardiac output, MAP, and coronary perfusion, may be a viable medium through which hemodynamic instability and cardiac stunning during HD can be prevented (Figure 3). By virtue of the fact that a progressive decline in cardiac output is known to correlate with a deterioration in coronary perfusion during HD [17], it would seem logical that an increase in cardiac output, achieved by intradialytic exercise, would enhance perfusion and reduce cardiac stunning. However, there is currently very little evidence to support this hypothesis. The acute physiological response to submaximal exercise in ESRD is poorly defined, particularly during HD (Table 1), presumably due to the challenges associated with collecting this data.

### 6.1. Potential Therapeutic Effects

Some indication of the acute physiological response to intradialytic exercise can be derived from limited existing data. Patients with ESRD are known to have a significantly impaired maximal functional capacity (~75% of normal), mediated by CKD maladaptive LV hypertrophy, loss of arterial compliance, and a blunted chronotropic response [12]. This disordered physiology would suggest that the acute cardiovascular response to submaximal exercise is also likely to differ from that of a healthy individual. In patients with ESRD, studies have identified an altered cardiovascular response to submaximal exercise performed off HD [61]. Heart rate and oxygen uptake (VO_2_) appear to be blunted (~10 & ~45%, respectively) in comparison to healthy individuals, but datasets are small and inconclusive. With exercise during HD, a significant increase in HR and BP was observed (~15 & ~13%, respectively) with 30 minutes of low to moderate intensity cycling when compared to standard HD without exercise [60]. A cardiovascular response to intradialytic exercise is, therefore, evident and a concomitant increase in cardiac output and coronary perfusion can be assumed but not confirmed. As such, intradialytic exercise may acutely aid the regulation of hemodynamic instability. Aerobic exercise during HD can also lead to greater solute removal (e.g., Urea, H+, and creatinine) [63, 64]. This increased dialysis efficacy (urea reduction rate & Kt/V) is thought to be a result of increased muscle blood flow and dilation of capillary tissue beds [65, 66]. This acute physiological response to intradialytic exercise may also help increase blood volume by inducing greater reuptake of blood from tissue [65, 66]. It is possible that this may contribute to hemodynamic stability and offset IDH [62]. In Figure 3, we propose a model by which the acute physiological response to intradialytic exercise may positively influence multiple mechanisms in the cascade of hemodynamic instability during HD.

### 6.2. Potential Negative Effects

Acute negative effects of intradialytic exercise must also be considered. Systolic blood pressure was shown to be lower at one hour after intradialytic exercise compared to HD without exercise [60]. Although patients were asymptomatic, and BP had normalized by the end of HD, there may be a risk of “rebound” hypotension associated with intradialytic exercise. Likewise, it has been speculated that intradialytic exercise may further exacerbate HD induced gastric ischemia via the redirection of blood flow from splanchnic tissue to more metabolically active tissue [16]. As with data supporting the potential acute effects of intradialytic exercise, studies evaluating harm are scarce. Indeed, longitudinal intradialytic exercise training studies, whilst not specifically assessing the acute physiological response, overwhelmingly support the safety of this intervention. A variety of training modalities (cycling, resistance exercise, and electrical muscle stimulation) have identified numerous benefits with a negligible complication rate [4, 59, 67–71]. Peak oxygen uptake, muscular strength, arterial compliance, inflammation, and QOL have all improved with training. On balance, therefore, it does seem plausible that, acutely, the physiological response to intradialytic exercise may have a beneficial effect on hemodynamics and coronary perfusion, thus mitigating IDH and cardiac stunning. Exercise also has a proven advantage over other treatments in that the benefits may extend not only to abrogating hemodynamic instability during HD, but also to the numerous, well defined, chronic physiological and psychosocial adaptations of cardiovascular and resistance training [4, 31, 71–75].

## 7. Conclusion

Haemodialysis, although essential for patient survival, can predispose patients to cerebral, splanchnic, and coronary ischemia due to compromised cardiovascular hemodynamics. Despite the availability of a number of therapeutic strategies to alleviate hemodynamic instability during HD, the widespread adoption of these treatments is prevented by medical complications, limited efficacy, and lack of good quality evidence. Intradialytic exercise may offer a solution to this treatment conundrum and may have the potential to succeed where medical therapies are sometimes subtherapeutic. By reducing IDH and increasing myocardial perfusion, intradialytic exercise may ameliorate acute HD related complications and have a meaningful effect on long-term cardiovascular risk and mortality. However, these potential mechanisms require further investigation to fully characterize the acute physiological response to intradialytic exercise. It is also possible that intradialytic exercise may exacerbate the acute hemodynamic instability associated with HD. Either way, whether therapeutic or nontherapeutic, experimentation in this area will provide preliminary evidence, not only to shape treatment, but ultimately to inform the development of safe and effective guidelines for intradialytic exercise. We propose that the acute physiological response to intradialytic exercise be investigated, with the specific intention of treating hemodynamic instability and cardiac stunning during HD.

## Figures and Tables

**Table 1:** Summary of relevant previous research investigating the acute physiological responses to exercise in end stage renal disease patients.

Study (year)	Subjects	Outcome measures	Exercise	Results	Significant findings	Limitations
Banerjee et al. (2004) [59]	*N* = 20, Group 1 (4 males, 6 females, age 37 ± 12), group 2: (6 males, 4 females, age 47 ± 24),	% RBV, CO (echo).	Cycling at 20% above predialysis HR for 10 mins during isovolumic HD.	Drop in RBV at the end of exercise (3.0 ± 0.8 vs. 2.2 ± 1.5%, *P* = 0.001), CO increased after both periods of exercise (4.5 ± 0.96 and 5.1 ± 1.1 versus 7.2 ± 2.1 and 7.9 ± 2.41 l/min; *P* < 0.001)	Fall in RBV occurred immediately after the onset of submax exercise during isovolumic HD CO increased but did not result in increased vascular resistance	Relatively young individuals, small cohort

Dungey et al. (2015) [60]	*N* = 15 HD (9 males, 6 females; age: 58 ± 11 y),	HR, BP, RPP. Markers of cardiac injury, inflammation, haematology, neutrophil degranulation	30 mins cycling at RPE 13 during HD.	Increase in HR (~15%) and BP (~13%) during exercise. 1 h after exercise SBP dropped below control SBP (106 ± 22 versus 117 ± 25, *P* = 0.04). No increase in inflammatory markers after exercise	Exercise placed an additional demand on the heart at a time when it is at an increased risk of myocardial stunning Markers of cardiac injury did not differ	Exercise stimulus not sufficient (21.5 ± 8.1 W), small cohort

Kettner et al. (1984) [61]	*N* = 8 HD (age 29.6 ± 3.6 years). Control: *N* = 6 HS (age 31 ± 2 years)	RER, VO_2_, HR, BP, adrenaline, noradrenaline, glucose, insulin, glucagon	Cycling at 50% VO_2_ max for 60 mins off HD	HR (~10%) and VO_2_ (~45%) blunted in HD patients. Depressed RER at rest in HD patients compared to controls (~0.75 versus ~0.85), increased adrenaline, noradrenaline, glucose, insulin, glucagon in HD patients compared to controls (*P* < 0.05)	VO_2_ lower in HD patients. Elevated plasma levels of hormones related to reduced renal clearance of active and inactive hormones	Small cohort

Ookawara et al. (2016) [62]	*N* = 12 HD (age 71.0 ± 3.1 years). Control: *N* = 12 (age 70.8 ± 2.4).	HR, BP, ΔBV.	Cycling at 10% higher HR then baseline during HD for 25 mins	Increase in HR during exercise (61.8 ± 3.1 versus 67.9 ± 3.7, *P* = 0.002). A ~1% increase in BV after exercise	Attenuation in ultrafiltration induced BV reduction at the end of HD via increased plasma refilling from the interstitium to the blood vessels	Low intensity of exercise, small cohort

*Notes. *RBV: relative blood volume; BV: blood volume; CO: cardiac output; RPP: rate pressure product; HD: haemodialysis; GFR: glomerular filtration rate; HS: healthy subjects; ESRD: end stage renal disease; Echo: echocardiogram; HR: heart rate; BP: blood pressure; SBP: systolic blood pressure; RPE: rating of perceived exertion; RER: respiratory exchange ratio.
